# QT Interval Prolongation in People Treated With Bedaquiline for Drug-Resistant Tuberculosis Under Programmatic Conditions: A Retrospective Cohort Study

**DOI:** 10.1093/ofid/ofab413

**Published:** 2021-08-01

**Authors:** Sharon Isralls, Kathy Baisley, Eric Ngam, Alison D Grant, James Millard

**Affiliations:** 1 London School of Hygiene and Tropical Medicine, London, United Kingdom; 2 Africa Health Research Institute, Durban, South Africa; 3 University of KwaZulu-Natal, Durban, South Africa; 4 Tuberculosis Centre, London School of Hygiene and Tropical Medicine, London, United Kingdom; 5 School of Laboratory Medicine and Medical Sciences, College of Health Sciences, University of KwaZulu-Natal, Durban, South Africa; 6 School of Public Health, University of the Witwatersrand, Johannesburg, South Africa; 7 Wellcome Trust Liverpool Glasgow Centre for Global Health Research, Liverpool, United Kingdom; 8 Institute of Infection and Global Health, University of Liverpool, Liverpool, United Kingdom

**Keywords:** bedaquiline, QT interval prolongation, tuberculosis

## Abstract

**Background:**

Bedaquiline has a black-box warning of the risk of arrhythmias and sudden death. This study aimed to determine the incidence of QTc prolongation and cardiac events in patients receiving bedaquiline for drug-resistant tuberculosis (DR-TB) under programmatic conditions.

**Methods:**

Retrospective cohort study of patients receiving bedaquiline at a DR-TB hospital in KwaZulu Natal, South Africa from September 2017 to February 2019. The primary outcome, a prolonged QT interval corrected using the Fridericia formula (QTcF), was defined as QTcF >500 ms, QTcF change >60 ms from baseline, or both.

**Results:**

Among 420 patients (66.2% male, median age 36 years), the median QTcF was 406.4 (interquartile range [IQR], 389.1–421.3) ms at baseline, increasing to 430.5 (IQR, 414.4–445.1) ms by 3 months and 434.0 (IQR, 419.0–447.9) ms at 6 months. Eighteen of 420 patients (4.3%) had a QTcF >500 ms and 110 of 420 patients (26.2%) had a QTcF change >60 ms. There were no recorded arrhythmias or cardiac deaths. Odds of prolonged QTcF were increased with concomitant azoles (adjusted odds ratio [aOR], 5.61 [95% confidence interval (CI), 2.26–13.91]; *P* < .001) and an inverse association with HIV-positive status (aOR, 0.34 [95% CI, .15–.75]; *P* = .008) and hypertension (aOR, 0.13 [95% CI, .02–.86]; *P* = .02). After prolongation, the QTcF declined to <500 ms, whether drugs were interrupted or not.

**Conclusions:**

We observed a modest prolongation of QTcF, maximal at week 15; there were no recorded arrhythmias or related deaths.

## Teaser

A modest QTcF prolongation, maximal at 15 weeks, was observed in patients receiving bedaquiline treatment for drug-resistant tuberculosis under programmatic conditions in KwaZulu-Natal, South Africa. There were no recorded arrhythmias or related deaths.

There were an estimated 465 000 incident cases of drug-resistant tuberculosis (DR-TB) in 2019, 14 000 of which were in South Africa [1]. Treatment for DR-TB has long relied on prolonged regimens of poorly efficacious, toxic drugs, with treatment success at around 50% globally [2, 3]. The advent of novel drugs such as bedaquiline, delamanid, and pretomanid and repurposing of drugs such as clofazimine and linezolid promise improved DR-TB treatment.

Bedaquiline belongs to a novel class of TB drugs, the diarylquinolines, and is associated with improved outcomes [4–6]. World Health Organization (WHO) DR-TB guidelines categorize bedaquiline as a group A drug to be included as part of all long regimens for DR-TB [7]. In South Africa, bedaquiline is used as part of a modified 9- to 12-month regimen [8]. As of June 2020, 29 193 patients in South Africa have received bedaquiline as part of their DR-TB therapy [9]. Bedaquiline has a favorable safety profile [10], but is known to prolong the QT interval on electrocardiogram (ECG). QT prolongation increases the risk of arrhythmias, specifically torsades de pointes [11]. In the phase 2b trial of bedaquiline (TMC207-C208), there was an excess of deaths in the bedaquiline arm (10/79 [13%]) compared to the placebo arm (2/81 [2%]) (*P* = .02 for difference) [12]. Although no association was found between these deaths and QT prolongation and most deaths occurred after bedaquiline had been completed [5], the United States Food and Drug Administration issued a black-box warning of a risk of QT prolongation, arrhythmias, and sudden death with bedaquiline [13, 14].

In subsequent trials (TMC207-C209) and programmatic evaluations, QT prolongation with bedaquiline has been confirmed [11, 15, 16]. However, the QT interval infrequently exceeded 500 ms, arrhythmias were rarely reported, and no episodes of torsades de pointes have been reported [5, 11]. There is a need for further data on the safety of bedaquiline in South Africa where it is used alongside clofazimine, a fluoroquinolone (typically levofloxacin), and often other drugs (eg, azoles), which also prolong the QT interval. The South African bedaquiline clinical access program, where bedaquiline was used in combination with clofazimine and fluoroquinolones in the majority of patients, reported low rates of QT prolongation. However, many of these patients had a “baseline” ECG performed while already taking moxifloxacin, making QT prolongation due to bedaquiline difficult to identify [2, 16, 17].

South African guidelines recommend performing an ECG at baseline, after 2 weeks, 4 weeks then monthly while on bedaquiline, or more frequently if additional QT-prolonging drugs are being taken [8]. If the QT interval corrected using the Fridericia formula (QTcF) is >470 ms but <500 ms, asymptomatic patients can be monitored with serial ECGs weekly until stable. If QTcF is >500 ms, all QT-prolonging drugs are to be stopped, followed by management of other causes of QTc prolongation, and 48 hours later, a repeat ECG is recommended [8]. Each clinic delivering DR-TB treatment requires an ECG machine and expertise to read the ECG. This is not always available at peripheral centers, imposes additional expense, and may act as a barrier to bedaquiline rollout in some settings [18]. Understanding the timing of QT prolongation and who is most at risk may help guide ECG monitoring requirements in South Africa.

We report the incidence of QTc prolongation and cardiac events for a large cohort of patients treated with a combination of DR-TB agents including bedaquiline under programmatic conditions in South Africa.

## METHODS

### Patient Selection

This was a retrospective cohort study at a specialist DR-TB hospital in eThekwini District, KwaZulu-Natal, South Africa. Consecutive patients initiating bedaquiline-based DR-TB therapy from September 2017 (the date from which bedaquiline was first available in the hospital) to February 2019 were identified using both the hospital register and the Electronic Drug-Resistant Tuberculosis Register (EDRWeb). The standard duration of bedaquiline therapy as per South African guidelines was 6 months [8].

### Inclusion and Exclusion Criteria

Patients were excluded if they were not treated with a bedaquiline-based DR-TB regimen, if they did not have a baseline ECG recorded within the 4 weeks prior to bedaquiline initiation, or if they did not have at least 1 follow-up ECG in the 6 months of bedaquiline treatment. Patients were eligible regardless of their background DR-TB regimen.

### Data Collection

A standardized data collection tool was developed using Open Data Kit [19]. We collected relevant baseline characteristics, including comorbidities, and follow-up data for the duration of bedaquiline therapy, including all other antituberculosis drugs and any interruptions in therapy. Electrolytes were measured, but not in a consistent manner, limiting analysis for other risk factors of torsades de pointes. Twelve-lead ECGs were reviewed by a clinician and heart rate, QT interval, and any abnormalities were recorded. The QT interval was corrected for heart rate using the Fridericia formula: $QTcF=\;QT/\sqrt[{3}]{RR}$. Significant QT interval prolongation was defined, in accordance with WHO guidelines [7], as a QTcF measurement >500 ms, or an increase of >60 ms from baseline.

### Statistical Analysis

We used Kaplan-Meier methods to estimate retention on bedaquiline treatment for 6 months. Person-time was calculated from the start of bedaquiline therapy until the earliest of stopping bedaquiline (either permanently or an interruption of treatment), death, loss to follow-up, or 183 days on treatment. Patients were censored when transferred out. Patients who were prescribed bedaquiline for <6 months were censored on the date of completion of therapy. Those who were still on treatment at the time of record review were administratively censored on the date of their last record. As a sensitivity analysis, patients who interrupted treatment temporarily were considered to be retained on treatment, and person-time was calculated ignoring the interruption.

We used random effects logistic regression to estimate odds ratios (ORs) and 95% confidence intervals (CIs) for the association of factors with the primary outcome of QTcF >500 ms or an increase of >60 ms from baseline at each visit. We restricted the analysis to the first 26 weeks of treatment with bedaquiline. We examined the change in QTcF over time using mixed-effects linear regression to account for the correlation of observations within patients. To allow for nonlinear effects of time, visit week was modeled using restricted cubic splines with 4 knots; this approach provides a flexible way to model the shape of the relationship of a continuous covariate with the outcome. We adjusted for age, sex, and visit week as a priori confounders; visit week was modeled using restricted cubic splines. Factors that were associated with the outcome at *P* < .20 after adjusting for age, sex, and week were included in a multivariable model; those that remained associated at *P* < .10 were retained. All analyses used Stata version 15 software.

### Patient Consent Statement

The study was approved by the research ethics committees of the University of KwaZulu-Natal (BE314/19) and the London School of Hygiene and Tropical Medicine (16481), and by the Department of Health of KwaZulu-Natal (KZ_201906_001). Due to the retrospective nature of this study, the requirement for written informed consent was waived.

## RESULTS

### Patient Enrollment

Among 450 patient records reviewed, 420 were eligible for inclusion. Reasons for exclusion were as follows: no ECG before bedaquiline treatment initiation (n = 23), no baseline ECG within 4 weeks of bedaquiline treatment initiation (n = 6), or no post–bedaquiline initiation ECG (n = 1).

### Baseline Characteristics

Among 420 patients, the median age was 36 (interquartile range [IQR], 29–44) years and 66.2% were male (Table 1). The majority (312/420 [74.3%]) were human immunodeficiency virus (HIV) positive, and 201 of 312 (64.4%) were taking antiretroviral therapy (ART) prior to starting bedaquiline. Median CD4 count at time of bedaquiline initiation was 241 cells/mm^3^. All 420 patients were prescribed bedaquiline with an optimized background regimen that included clofazimine and levofloxacin. Forty-seven of 420 (11.2%) were on moxifloxacin at the time of baseline ECG measurement; 46 of these were subsequently converted to levofloxacin.

**Table 1.** Demographic and Clinical Characteristics of 420 Patients at Baseline

Characteristic	No. (%) or Median (IQR)
Age, y, median (IQR)	36 (29–45)
Sex, male	278 (66.2)
Non-smoker	261 (62.4)
HIV status	
Positive	312 (74.3)
On ART before DR-TB treatment	201 (64.4)
CD4 count, cells/mm^3^, median (IQR) (n = 309)	175 (65–376)
Hypertension (yes)	32 (7.6)
Diabetes (yes)	19 (4.5)
Hypothyroidism	
Yes	95 (22.6)
Unknown	325 (77.4)
Epilepsy (yes)	6 (1.4)
QTcF at baseline, ms	
>450	15 (3.6)
>500	2 (0.5)
QTcF at baseline, ms, median (IQR)	406.4 (389.1–421.3)
Optimized background DR-TB regimen	
Including clofazimine	419 (99.8)
Including levofloxacin	419 (99.8)
Third agent in optimized ART regimen	
Nevirapine	208/309 (67.3)
Lopinavir/ritonavir	103/309 (33.3)
Atazanavir/ritonavir	2/309 (0.6)
Raltegravir	1/309 (0.3)
Dolutegravir	1/309 (0.3)

Abbreviations: ART, antiretroviral therapy; DR-TB, drug-resistant tuberculosis; HIV, human immunodeficiency virus; IQR, interquartile range; QTcF, Fridericia-corrected QT interval.

### Duration of Bedaquiline Treatment

**Figure 1.**
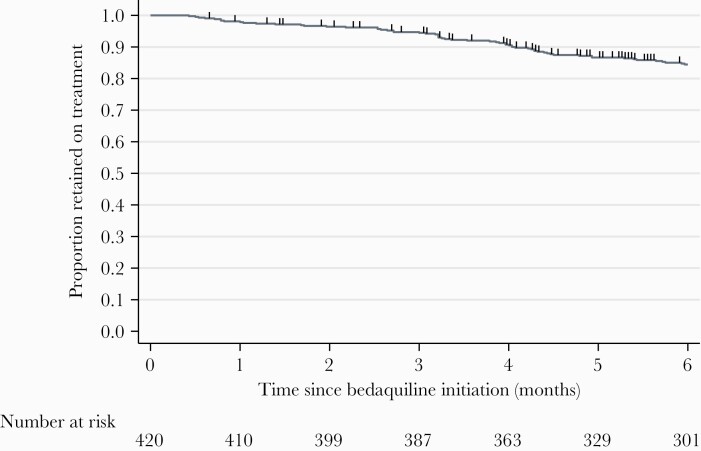
Kaplan-Meier survival estimate of remaining on bedaquiline treatment at 6 months. Vertical marks show censoring times.

Median time on bedaquiline among the 420 patients was 6.0 (IQR, 5.0–6.0) months. The Kaplan-Meier estimate for retention on bedaquiline treatment at 6 months, not discounting treatment interruptions, was 84.5% (Figure 1). At completion of the data collection period, 23 (5.5%) patients were still undergoing bedaquiline treatment, and 27 (6.4%) patients had transferred to another hospital before the end of treatment. Of the 62 (14.8%) patients who were not retained on bedaquiline treatment, 17 of 62 (27.4% [4.0% of all patients]) were lost to follow-up, 12 (19.4% [or 2.9% of all patients]) died, and 7 (11.3% [1.7% of all patients]) stopped treatment. Twenty-six of 62 patients (41.9% [6.2% of all patients]) temporarily interrupted treatment; median time to restart bedaquiline was 28 (IQR, 15–54) days. In the sensitivity analysis discounting treatment interruptions, 90.4% of patients were retained on bedaquiline treatment at 6 months.

### Concomitant Medication

Two hundred one of 312 (64.4%) patients living with HIV were already on ART prior to DR-TB therapy: this increased to 99% (309/312) during bedaquiline DR-TB therapy treatment. Median time to ART initiation was 24 (IQR, 17–34) days after bedaquiline treatment initiation. All ART regimens included 2 nonnucleoside/nucleotide reverse transcriptase inhibitors; the third agent was nevirapine in 208 of 309 (67.3%), lopinavir/ritonavir in 103 (33.3%), atazanavir/ritonavir in 2, and raltegravir and dolutegravir in 1 each.

### QTc Interval Measures and Clinical Events

The median QTcF interval at baseline was 406.4 (IQR, 389.1–421.3) ms. Eighteen patients (4.3%) had a recorded QTcF >450 ms at baseline, of whom 6 (33%) had a repeat baseline ECG; QTcF remained >450 ms in all cases. Two patients (11%) had a QTcF >500 ms at baseline, both of whom commenced therapy without a repeat predose ECG. A median of 8 ECGs (IQR, 7–9) per patient were performed during the 6 months after starting bedaquiline. During these 6 months, 18 patients (4.3%) experienced at least 1 episode of QTcF >500 ms (24 episodes), and 110 patients (26.2%) had a change of >60 ms from baseline (253 episodes). Only 2 of these patients were among the 18 patients with QTcF >450 ms at baseline (11%), compared to 27% of patients with baseline QTcF <450 ms (*P* = .13).

**Figure 2.**
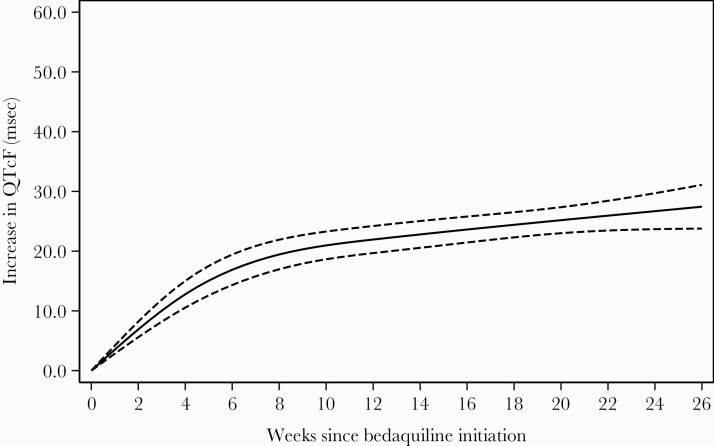
Mean increase in Fridericia-corrected QT interval (QTcF) from baseline over time, modeled using restricted cubic splines with 4 knots in a mixed-effect linear regression. Solid line is predicted mean increase in QTcF; dashed lines are 95% confidence intervals.

**Figure 3.**
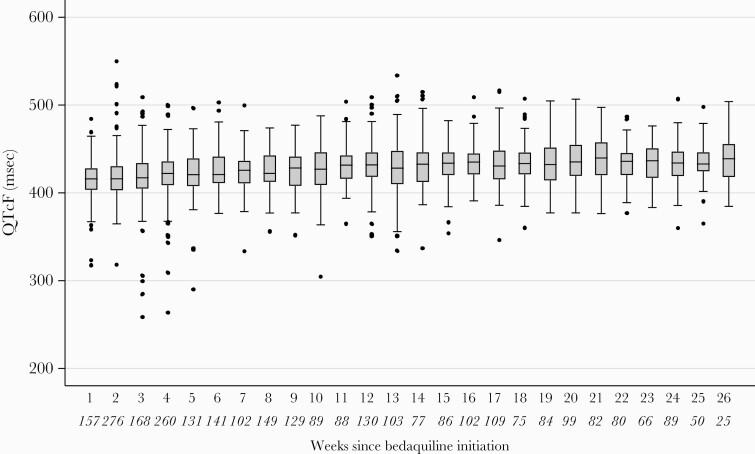
Box and whisker plots of Fridericia-corrected QT interval (QTcF) values over time, during the 6 months after bedaquiline initiation. The vertical line within the box indicates the median, the boundaries of the box indicate the interquartile range (25th and 75th percentiles), and the whiskers indicate values that are within 1.5 times the interquartile range above the 75th percentile, or 1.5 times the interquartile range below the 25th percentile. Values outside that range are plotted as individual points. The number of electrocardiograms at each timepoint are indicated in italics below the x-axis.

**Figure 4.**
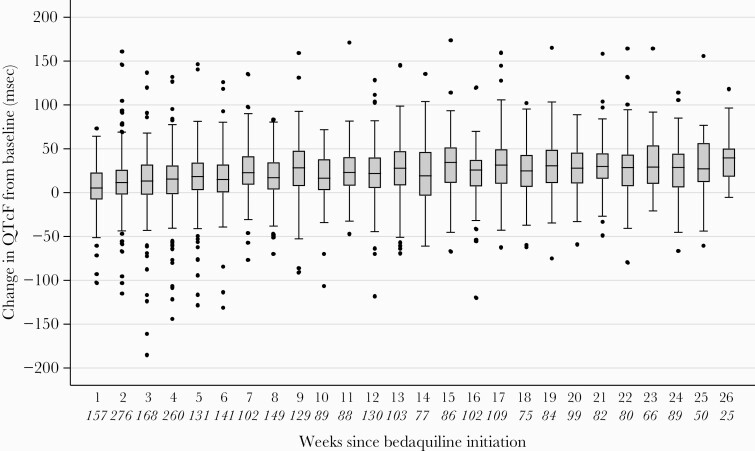
Box and whisker plots of change in Fridericia-corrected QT interval (QTcF) from baseline over time, during the 6 months after bedaquiline initiation. The vertical line within the box indicates the median, the boundaries of the box indicate the interquartile range (25th and 75th percentiles), and the whiskers indicate values that are within 1.5 times the interquartile range above the 75th percentile, or 1.5 times the interquartile range below the 25th percentile. Values outside that range are plotted as individual points. The number of electrocardiograms at each timepoint are indicated in italics below the x-axis.

When modeled as a function of time, the largest increase in QTc occurred in the first 6 weeks after bedaquiline initiation, after which the rate of increase leveled off (Figure 2). After 3 months on bedaquiline therapy, median QTc was 430.5 (IQR, 414.4–445.1) ms and after 6 months it was 434.0 (IQR, 419.0–447.9) ms (Figure 3). Median change in QTcF from baseline after 3 months on bedaquiline therapy was 24.5 (IQR, 6.2–43.4) ms and after 6 months it was 29.5 (IQR, 9.6–47.2) ms (Figure 4). Sixty-five patients (15.5%) were on treatment with an azole at the time of ECG measurement (202 visits). There were 42 occurrences in 14 patients of QTcF change >60 ms while taking concomitant azoles, among which there were 5 occurrences in 1 patient of QTcF >500 ms.

There were no clinically significant cardiovascular events or arrhythmias recorded. Twelve patients (2.7%) died, none of whom had a recorded QTcF >500 ms and all of whom had ECGs performed at timepoints consistent with the guidance. The cause of death was not recorded in the medical records.

### Management of QTc >500 ms and QTcF Change >60 ms

No action was taken if there was a QTcF change >60 ms above baseline (253 episodes). The 24 episodes of QTcF >500 ms are outlined in Table 2; the QT interval declined after prolongation without clinical sequelae, whether bedaquiline, levofloxacin, or clofazimine were interrupted or not. QTcF returned to <500 ms in all cases irrespective of management. There were too few data for formal analysis.

**Table 2.** Drug Management in Response to Fridericia-Corrected QT Interval >500 ms During 6 Months After Bedaquiline Initiation

Patient ID	QTcF >500 ms				Follow-up ECG #1			Follow-up ECG #2		
	QTcF, ms	Days Since BDQ Initiation	MDR-TB Regimen at Time of ECG	Drugs Interrupted After ECG	QTcF, ms	Days Since QTcF >500 ms	Drug Management After Follow-up ECG #1	QTcF, ms	Days Since QTcF >500 ms	Drug Management After Follow-up ECG #2
B197	500.9	12	BDQ, CFZ, LFX	BDQ, CFZ, LFX	416.7	2	CFZ interrupted	299.2	6	No further changes
B434	549.9	12	BDQ, CFZ, LFX	No change	475.1	7	No change	426.2	9	No change
B434	500.7	82	BDQ, CFZ, LFX	No change	533.7	7	No change	…	…	…
B434	533.7	89	BDQ, CFZ, LFX	BDQ, CFZ, LFX	508.8	21	BDQ resumed	…	…	…
B434	508.8	110	BDQ	No change	516.7	8	No change			
B434	516.7	118	BDQ	No change	471.9	4	No change	484.4	8	None
B010	521.4	13	BDQ, CFZ, LFX	No change	467.0	8	No change	457.7	14	No change
B010	515.3	120	BDQ, CFZ, LFX	No change	472.4	42	No change	473.9	56	No change
B319	523.8	15	BDQ, CFZ, LFX	BDQ, CFZ, LFX	450.5	7	CFZ interrupted	446.1	15	No further changes
B051	509.0	21	BDQ, CFZ, LFX	No change	492.4	1	BDQ, CFZ, LFX interrupted	460.0	6	No further changes
B127	502.9	39	BDQ, CFZ, LFX	No change	427.0	8	CFZ interrupted	403.4	21	No further changes
B223	503.8	80	BDQ, CFZ, LFX	No change	496.2	19	No change	484.0	38	No change
B026	509.0	83	BDQ, CFZ, LFX	No change	436.9	1	No change	440.1	8	No change
B333	510.1	90	BDQ, CFZ, LFX	BDQ, CFZ, LFX	479.6	28	BDQ, CFZ, LFX interrupted	450.2	38	No further changes
B238	505.3	93	BDQ, CFZ, LFX	No change	461.9	11	No change	455.0	14	No change
B107	506.8	98	BDQ, CFZ, LFX	CFZ	460.8	8	CFZ interrupted	429.5	56	No further changes
B214	510.7	99	BDQ, CFZ, LFX	LFX	466.9	7	LFX interrupted	485.9	25	No further changes
B283	515.1	100	BDQ	No change	449.0	6	No change	444.8	20	No change
B398	507.0	126	BDQ, CFZ, LFX	BDQ, CFZ, LFX	480.8	9	BDQ, CFZ, LFX interrupted	468.7	16	No further changes
B327	504.7	130	BDQ, CFZ, LFX	BDQ, CFZ	497.4	14	No change	…	…	…
B327	507.3	167	Rx completed	NA	456.4	104	No change	…	…	…
B408	506.7	141	BDQ, CFZ, LFX	No change	479.9	28	No change	…	…	…
B037	506.3	170	BDQ, CFZ, LFX	No change	498.1	6	No change	…	…	…
B208	504.0	180	BDQ, CFZ, LFX	No change	No follow-up ECG					

Abbreviations: BDQ, bedaquiline; CFZ, clofazimine; ECG, electrocardiogram; LFX, levofloxacin; MDR-TB, multidrug-resistant: NA, not applicable; QTcF, Fridericia-corrected QT interval; Rx, prescription.

### Predictors of QTcF Prolongation

**Figure 5.**
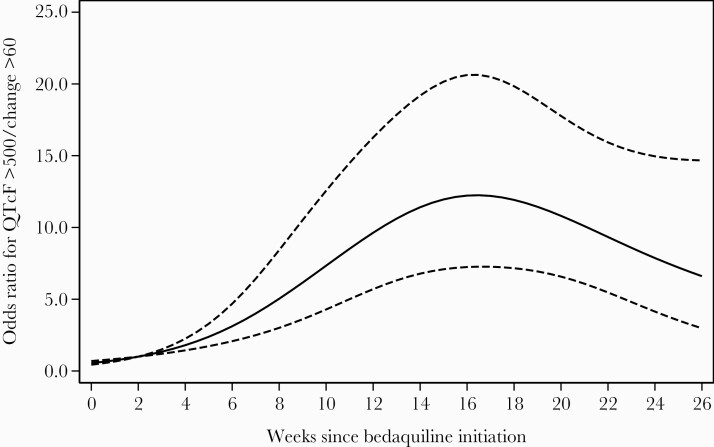
Association of Fridericia-corrected QT interval (QTcF) prolongation (QTcF >500 ms or change from baseline >60 ms) with time since bedaquiline start, modeled using restricted cubic splines with 4 knots in a random-effect logistic regression model. Solid line is estimated odds ratio (OR) for QTcF prolongation; dashed lines are 95% confidence intervals. Week 2 was used as the reference to calculate the ORs.

We recorded 254 episodes of QTcF >500 ms or change from baseline >60 ms among 111 patients during the 6 months of bedaquiline therapy. The odds of this combined outcome of QTcF prolongation increased over time and were highest after 15 weeks on treatment (Figure 5). In the final adjusted analysis, there was strong evidence of an increased odds of QT prolongation with concomitant azole antifungals (adjusted OR [aOR], 5.61 [95% CI, 2.26–13.91]; *P* < .001); median time on azoles was 14 (IQR, 14–14) days and there was strong evidence of an inverse association with HIV-positive status (aOR, 0.34 [95% CI, .15–.75]; *P* = .008) and hypertension (aOR, 0.13 [95% CI, .02–.86]; *P* = .02) (Table 3). There was also weak evidence of an inverse association with diabetes (aOR, 0.11 [95% CI, .01–1.31]; *P* = .11). There was no evidence of an association with QTc prolongation in 12 of 420 (2.9%) patients treated concomitantly with bedaquiline and delamanid (aOR, 1.17 [95% CI, .30–4.58]; *P* = .82).

**Table 3.** Factors Associated With Combined Outcome of Fridericia-Corrected QT Interval >500 ms and/or Change From Baseline >60 ms at Each Visit, During 6 Months After Bedaquiline Initiation

Characteristic	Patients With QTcF >500 ms or Change >60 ms/Total Patients (%) (N = 420 Patients)	ECGs With QTcF >500 ms or Change >60 ms/Total ECGs (%) (n = 2955 ECGs)	Crude OR (95% CI)	Adjusted OR (95% CI)^a^
Age group, y			*P* = .83	*P* = .15
<30	27/119 (22.7)	65/781 (8.3)	1	1
30–49	69/247 (27.9)	160/1769 (9.0)	1.23 (.60–2.53)	1.91 (.85–4.29)
≥50	15/54 (27.8)	29/405 (7.2)	1.03 (.36–2.93)	2.90 (.85–9.91)
Sex			*P* > .99	*P* = .36
Male	75/278 (27.0)	169/1982 (8.5)	1	1
Female	36/142 (25.4)	85/973 (8.7)	1.00 (.52–1.92)	1.40 (.68–2.88)
HIV positive			*P* = .18	*P* = .008
No	36/110 (32.7)	75/788 (9.5)	1	1
Yes	75/310 (24.2)	179/2167 (8.3)	0.63 (.32–1.24)	0.34 (.15–.75)
Hypertension			*P* = .02	*P* = .02
No	108/388 (27.8)	249/2743 (9.1)	1	1
Yes	3/32 (9.4)	5/212 (2.4)	0.14 (.03–.73)	0.13 (.02–.86)
Diabetes			*P* = .05	*P* = .06
No	109/401 (27.2)	252/2823 (8.9)	1	1
Yes	2/19 (10.5)	2/132 (1.5)	0.12 (.01–1.03)	0.11 (.01–1.31)
Levofloxacin^b^			*P* = .06	*P* = .37
No	1/1 (100)	22/133 (16.5)	1	1
Yes	110/419 (26.3)	232/2822 (8.2)	0.48 (.23–1.04)	0.68 (.29–1.58)
Clofazamine^b^			*P* = .07	*P* = .59
No	1/2 (50.0)	25/123 (20.3)	1	1
Yes	110/418 (26.3)	229/2832 (8.1)	0.51 (.25–1.06)	0.80 (.36–1.77)
Moxifloxacin^b^			*P* = .72	*P* = .44
No	110/413 (26.6)	253/2924 (8.7)	1	1
Yes	1/7 (14.3)	1/31 (3.2)	0.54 (.02–15.44)	0.25 (.01–10.82)
Delamanid^b^			*P* = .26	*P* = .82
No	107/408 (26.2)	246/2889 (8.5)	1	1
Yes	4/12 (33.3)	8/66 (12.1)	2.08 (.59–7.35)	1.17 (.30–4.58)
Lopinavir/ritonavir^b^			*P* = .96	*P* = .53
No	83/320 (25.9)	200/2343 (8.5)	1	1
Yes	28/100 (28.0)	54/612 (8.8)	1.02 (.51–2.01)	0.79 (.37–1.68)
Metoclopramide^b^			*P* = .02	*P* = .42
No	95/353 (26.9)	250/2832 (8.8)	1	1
Yes	16/67 (23.9)	4/123 (3.3)	0.23 (.07–.80)	0.59 (.16–2.22)
Amitriptyline			*P* = .84	*P* = .96
No	110/416 (26.4)	253/2946 (8.6)	1	1
Yes	1/4 (25.0)	1/9 (11.1)	0.63 (.01–48.05)	0.89 (.01–156.80)
Haloperidol^b^			*P* = .57	*P* = .69
No	110/411 (26.8)	253/2944 (8.6)	1	1
Yes	1/9 (11.1)	1/11 (9.1)	2.17 (.15–31.94)	1.79 (.10–31.32)
Cotrimoxazole^b^			*P* = .99	*P* = .37
No	38/120 (31.7)	78/943 (8.3)	1	1
Yes	73/300 (24.3)	176/2012 (8.7)	1.00 (.54–1.83)	1.75 (.51–6.06)
Azole antifungals^b^			*P* = .01	*P* < .001
No	89/355 (25.1)	212/2753 (7.7)	1	1
Yes	22/65 (33.8)	42/202 (20.8)	2.82 (1.26–6.30)	5.61 (2.26–13.91)

Abbreviations: CI, confidence interval; ECG, electrocardiogram; HIV, human immunodeficiency virus; OR, odds ratio; QTcF, Fridericia-corrected QT interval.

^a^Adjusted for age, sex, and visit week (a priori), HIV status, hypertension, diabetes, and azole antifungals.

^b^Taking the medication concomitant with bedaquiline at the time of the ECG measurement.

## DISCUSSION

This study among patients taking bedaquiline under programmatic conditions demonstrated a progressive increase in QTcF from baseline, with a cumulative incidence of QTcF >500 ms or QTcF >60 ms from baseline of 4.3% and 26.2%, respectively, over 6 months of bedaquiline therapy, and maximum odds for the combined outcome at 15 weeks of bedaquiline therapy. However, we found no evidence of clinical adverse events associated with these changes.

Under randomized controlled trial conditions and in combination with ofloxacin or levofloxacin, but without concomitant clofazimine, the mean increase in QTc is in the order of 11.9–15 ms, with an incidence of QTcF >500 ms of ≤1% [10, 12, 20]. However, several other observational studies have reported a higher average increase in QTcF (31.9 ms median maximum increase [21] and 49 ms mean change from baseline [21]) and a higher incidence of QTcF >500 ms (11% [21] and 15% [22], respectively). There are several potential explanations for the higher incidence of QT prolongation seen in our study and other observational cohorts. Clofazimine monotherapy increases the QT interval and in the original open-label trial of bedaquiline (C209), the mean increase in QTcF at week 24 was far higher in participants taking vs not taking clofazimine (31.9 ms vs 12.3 ms) [15, 23]. Several other cohorts have included patients taking concomitant clofazimine, but to our knowledge this is the largest study reporting QT prolongation in a cohort where concomitant clofazimine administration was universal. The largest comparable study in South Africa among 200 patients receiving bedaquiline, clofazimine, and fluoroquinolones reported a median increase in QTc of 11 (IQR, -6 to 27) ms and 5 cases of QTcF >500 ms, but many patients were already on moxifloxacin when “baseline” ECG monitoring was performed, making the results difficult to interpret [2, 16, 17]. Participants in the bedaquiline licensing trials were excluded if they had a QTcF >450 ms at baseline or were taking other drugs likely to prolong the QT interval or to lead to drug–drug interactions (including antiretrovirals) [12, 15]. Our study was designed to represent patients being treated for DR-TB under programmatic conditions and included >90% of patients started on bedaquiline in this facility during the study time period. Many were taking antiretroviral and other concomitant drugs. Of note, 18 patients (4.3%) were initiated on bedaquiline with a baseline QTcF >450 ms, and 2 patients (0.5%) with a baseline QTcF >500 ms. It is therefore probable that patients in our study, like others in DR-TB programs, had higher baseline risk for QT prolongation than those recruited in clinical trials. It was reassuring that we found no evidence of clinical consequences of QT prolongation, consistent with a growing body of literature suggesting that arrhythmias and sudden cardiac death are rare among people taking bedaquiline under routine conditions [6, 11, 16]. QT measurements are known to vary by up to 100 ms within an individual patient in a single day and this variability is influenced by factors such as patient positioning, time since last meal, and time of the day [24, 25]. ECG monitoring in clinical trials typically follows a strict protocol to reduce variability. ECG monitoring in a “real world” setting may overestimate the incidence of episodes of QT prolongation based on single ECGs. We argue that it is important to establish the incidence of QT prolongation identified using routine monitoring, as this is what programmatic clinical decisions will be based on.

There are a number of established risk factors that predispose to QTc prolongation, notably female sex, age, electrolyte disturbance, and certain drug classes [26]. In our study, azole antifungals increased the odds of QTc prolongation; however, only 1 patient had a documented QTcF >500 ms. We found no evidence of an association with QTc prolongation and delamanid coadministration, but this was limited to observation of 12 patients. Ninety-nine percent of the HIV-positive patients in our cohort received ART. Surprisingly, being HIV positive was inversely associated with QTc prolongation, but there may be residual confounding. The interim analysis of 91 patients in the bedaquiline clinical access program cohort found no association with QTcF prolongation and positive HIV status [16]. Increased incidence of QT prolongation has been described among HIV-positive people receiving efavirenz [26, 27]; in line with national guidelines, no patients in our cohort were taking efavirenz [8, 26]. The data are reassuring for the use of bedaquiline among patients taking ART.

In our study, in line with guidelines [8], we observed no change in management if QTcF increased by 60 ms. Management in response to patients with QTcF >500 ms varied based on physician discretion, as shown in Table 2. The rationale was not clearly documented in medical records, a limitation of this retrospective study. In all patients, the QTc reverted to <500 ms irrespective of whether or not drugs were stopped. Drug interruption introduces the risk of poor outcomes and resistance development [28, 29]. The optimal management of QTc prolongation during bedaquiline therapy is yet to be defined.

In this cohort the QTcF increased most steeply from baseline in the first 4–6 weeks, then continued to increase more slowly until the end of bedaquiline therapy. Similarly, in the C208 licensing trial, the maximal increase in QTcF was observed in weeks 1–8 [15, 30]. Notably, the odds of having the combined outcome (QTcF >500 ms or a change in QTcF >60 ms) was highest at week 15. However, incidents of QTcF >500 ms were detected outside this higher-risk period and in general, our data support the current recommendations for ECG monitoring in South Africa. A limitation of this study is its retrospective nature and sample size. There is still a need for a prospective study with standardized ECG monitoring among patients receiving bedaquiline in routine care in the context of the 9- to 12-month regimen in South Africa and other settings. This would allow us to define patients most at risk for QTc prolongation as well as better define the higher-risk period, informing ECG guidelines.

In conclusion, among a cohort of patients receiving bedaquiline alongside clofazimine and levofloxacin for treatment of DR-TB in a routine setting in South Africa, we observed a modest prolongation of the QTc interval, with the greatest QTcF increase in the first 4–6 weeks and the highest odds of combined outcome at week 15. We observed no arrhythmias or attributable deaths, adding to data suggesting that bedaquiline is safe under programmatic conditions.

## Supplementary Data

Supplementary materials are available at *Open Forum Infectious Diseases* online. Consisting of data provided by the authors to benefit the reader, the posted materials are not copyedited and are the sole responsibility of the authors, so questions or comments should be addressed to the corresponding author.

## Notes

***Author contributions.*** Study conceptualization: S. I., J. M., and A. D. G. Data collection: S. I. and J. M. Data analysis: S. I. and K. B. Data interpretation: S. I., J. M., K. B., and A. D. G. Writing: S. I. and J. M. Review and approval of manuscript: S. I., J. M., K. B., E. N., and A. D. G.

***Acknowledgments.*** The authors thank all of the staff at the DR-TB site, with special mention to the medical records department. Thank you to Aruna Sevakram from the Africa Health Research Institute for administrative support.

***Financial support.*** This work was supported in part by the Wellcome Trust (grant number 201433/A/16/A; and 203919/Z/16/Z to J. M.). For the purpose of open access, the author has applied a Creative Commons public copyright license to any author-accepted manuscript version arising from this submission.

***Potential conflicts of interest.*** All authors: No reported conflicts of interest.
